# Understanding the extent of economic evidence usage for informing policy decisions in the context of India’s national health insurance scheme: Ayushman Bharat Pradhan Mantri Jan Aarogya Yojana (PM-JAY)

**DOI:** 10.1136/bmjgh-2024-015079

**Published:** 2024-06-10

**Authors:** Deepshikha Sharma, Akashdeep Singh Chauhan, Lorna Guinness, Abha Mehndiratta, Anamika Dhiman, Malkeet Singh, Shankar Prinja

**Affiliations:** 1 Department of Community Medicine and School of Public Health, Post Graduate Institute of Medical Education and Research (PGIMER), Chandigarh, India; 2 Center for Global Development, London, UK

**Keywords:** Health economics, Health insurance, Health policies and all other topics, Health services research, India

## Abstract

**Introduction:**

Ayushman Bharat Pradhan Mantri Jan Aarogya Yojana (PM-JAY) is one of the world’s largest tax-funded insurance schemes. The present study was conducted to understand the decision-making process around the evolution (and revision) of health benefit packages (HBPs) and reimbursement rates within PM-JAY, with a specific focus on assessing the extent of use of economic evidence and role of various stakeholders in shaping these policy decisions.

**Methods:**

A mixed-methods study was adopted involving in-depth interviews with seven key stakeholders involved in HBP design and reimbursement rates decisions, and a survey of 80 government staff and other relevant stakeholders engaged in the implementation of PM-JAY. The data gathered were thematically analysed, and a coding framework was developed to explore specific themes. Additionally, publicly available documents were reviewed to ensure a comprehensive understanding of the decision-making processes.

**Results:**

Findings reveal a progressive transition towards evidence-based practices for policy decisions within PM-JAY. The initial version of HBP relied heavily on key criteria like disease burden, utilisation rates, and out-of-pocket expenditures, along with clinical opinion in shaping decisions around the inclusion of services in the HBP and setting reimbursement rates. Revised HBPs were informed based on evidence from a national-level costing study and broader stakeholder consultations. The use of health economic evidence increased with each additional revision with consideration of health technology assessment (HTA) evidence for some packages and reimbursement rates based on empirical cost evidence in the most recent update. The establishment of the Health Financing and Technology Assessment unit further signifies the use of evidence-based policymaking within PM-JAY. However, challenges persist, notably with regard to staff capacity and understanding of HTA principles, necessitating ongoing education and training initiatives.

**Conclusion:**

While substantial progress has been made in transitioning towards evidence-based practices within PM-JAY, sustained efforts and political commitment are required for the ongoing systematisation of processes.

**WHAT IS ALREADY KNOWN ON THIS TOPIC**

- Ayushman Bharat Pradhan Mantri Jan Aarogya Yojana (PM-JAY) stands as one of the world’s largest national tax-based health insurance schemes. This scheme grants access to secondary and tertiary care by providing coverage for various medical and surgical procedures (that require hospitalisation or day care). These procedures are provided in the form of packages comprising of a list of various services, including diagnostics (pathology or radiology tests), curative interventions (surgical, medical, radiotherapy, etc), hospitalisation or day care, follow-up care, etc, required for treating a particular disease or a medical condition. There are over 1900 packages which are currently being covered under PM-JAY and together these 1900 packages are termed as ‘health benefit packages’ (HBPs) or ‘list of HBPs’.
- Since its inception in 2018, the scheme has notably revised what is included in the HBPs on an annual basis. While the use of economic evidence for reimbursement decisions is well established in high-income countries, the extent to which such evidence is used to shape decisions under PM-JAY has not been thoroughly documented.

**WHAT THIS STUDY ADDS**

- This provides a classic case study of PM-JAY using a stepwise approach towards more evidence-based policymaking, with expert opinion being supplemented with empirical evidence. The study highlights the potential barriers and challenges, in the form of lack of availability of high-quality and timely health technology assessment (HTA) evidence, and inadequate capacity to understand the use of HTA evidence in low/middle-income countries, faced during incorporating HTA or economic evidence into policy-level decision-making. Efforts like the establishment of the Health Financing and Technology Assessment unit further signify dedicated efforts to enhance evidence-based approaches in future revisions.

**HOW THIS STUDY MIGHT AFFECT RESEARCH, PRACTICE OR POLICY**

- The study offers lessons learnt and insights into initiating evidence-based decision-making processes and progressively enhancing their use. The identification of key elements, including routine cost data systems, HTA evidence, and unwavering political commitment, underscores the need for ongoing efforts to systematically advance these processes, contributing to the realisation of Universal Health Coverage.

## Introduction

India is undergoing a significant transition in policy decisions around purchasing and provision of healthcare services. The healthcare system in India is a mix of public and private providers. The public healthcare system is primarily managed by the government and consists of subcentres, primary healthcare centres, community health centres, district hospitals and tertiary care hospitals, which provide a range of outpatient and inpatient services including promotive, preventive, curative and rehabilitative care.^1^ Services provided by these public institutions are often subsidised for the patients. The private healthcare sector (including not-for-profit facilities) in India is extensive and diverse, ranging from small clinics to large corporate hospitals.^2^ Those who can afford to pay or with some health insurance mechanisms are able to access private healthcare.

While health insurance coverage is not universal in India, it is increasingly becoming popular. Existing health insurance schemes aim to cover approximately 70% of the population, although actual coverage is lower due to challenges in enrolling eligible individuals.^3^ Catering to different population segments, these schemes are broadly categorised into four main types, depending on the source of financing, that is, publicly funded health insurance, social health insurance (targeting specific groups such as central government employees, employees working in factories and business establishments, railway employees, ex-servicemen, etc),^4^ community-based health insurance (targeting specific communities or groups)^5^ and various voluntary private health insurance schemes. Each of these schemes offers different health benefit packages (HBPs) that include a carefully curated list of benefits across a wide array of healthcare procedures and services depending on the type of healthcare (ie, outpatient, inpatient, surgical, medical, etc) included in the mandate of the insurance scheme.^6–8^

Publicly funded health insurance in India includes the centrally sponsored Ayushman Bharat Pradhan Mantri Jan Aarogya Yojana (PM-JAY)—the largest national tax-funded health insurance scheme in the world launched in 2018, and various state-level individual health insurance schemes —known as extension schemes.^3^ These schemes provide health insurance cover to the poor households and the informal sector workforce, providing treatment coverage for conditions requiring secondary or tertiary care hospitalisation or day care. Before the introduction of the PM-JAY, various health insurance schemes such as Rashtriya Swasthya Bima Yojana (RSBY), Central Government Health Scheme (CGHS), Employees’ State Insurance Scheme (ESIS) and state-funded extension schemes ran in parallel with their own distinct HBPs. These schemes vary in the extent of coverage provided, with CGHS and ESIS covering both outpatient as well as inpatient services, while the RSBY covered only inpatient services. The launch of PM-JAY subsumed the RSBY scheme with the development of a more comprehensive HBP list known as HBP 1.0 of PM-JAY. Specifically, the PM-JAY provides cashless treatment for medical and surgical procedures requiring inpatient hospitalisation. These procedures are provided in the form of packages consisting of various services, including diagnostics (pathology or radiology tests), curative interventions (surgical, medical, radiotherapy, etc), hospitalisation or day care, follow-up care (drugs up to 15 days), etc, required for treating a particular disease or a medical condition. There are over 1900 packages which are currently being covered under PM-JAY and together these packages are termed as ‘HBP’ or the ‘HBP list’. While it is envisioned that future reforms could consider aligning all major health insurance schemes in India, currently, these are operating concurrently, each having its own HBP list and reimbursement rates, specific to the insurance scheme.

The PM-JAY is being implemented by the National Health Authority (NHA) which is responsible for ‘the overall vision and stewardship for design, roll-out, implementation and management of PM-JAY across the country’. This involves ‘formulation of PM-JAY policies, development of operational guidelines, implementation mechanisms, coordination with state governments, monitoring and oversight of PM-JAY among others’.^9^ In addition, State Health Agencies (SHAs) have been set up across states of India with full operational autonomy for implementing this scheme under the guidance of the NHA.^9^

**Figure 1:**
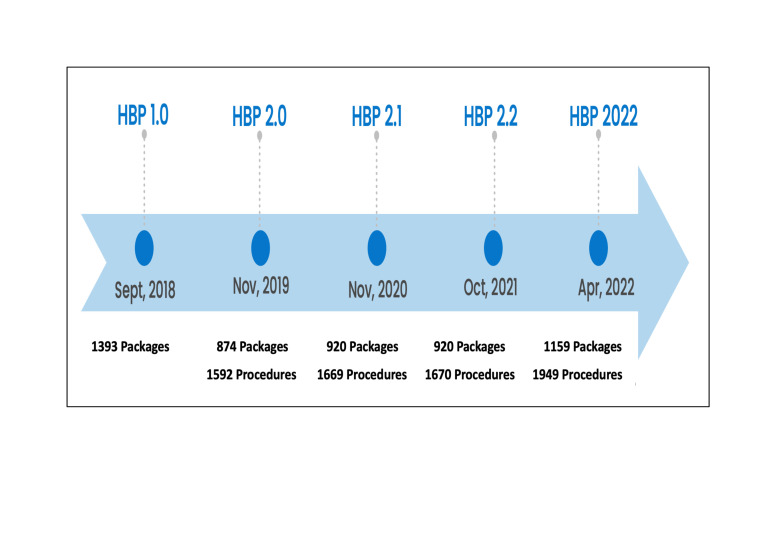
Evolution of Health Benefit Packages (HBPs) under Ayushman Bharat Pradhan Mantri Jan Aarogya Yojana.

An important aspect of the PM-JAY scheme and responsibility of the NHA is the regular update of the HBP list and setting appropriate reimbursement rates.^9^ Since the inception of the PM-JAY, the HBP list and reimbursement rates have been revised four times. HBP 1.0 was introduced in September 2018, and was followed by a major revision to HBP 2.0 in November 2019, with minor updates to HBP 2.1 in November 2020 and HBP 2.2 in October 2021. The next major revision was to HBP 2022, in April 2022 (figure 1). Decisions around the HBP design and setting reimbursement rates for schemes like PM-JAY should consider using evidencebased on locally generated data. This approach not only fosters accountability but also ensures the incorporation of robust estimates into the decision-making process. By relying on data specific to the region, policymakers can make more informed choices, by aligning more closely with the unique needs and dynamics of the local population, thereby contributing to the overall success and better sustainability of health programmes.

There is a limited body of global literature on HBP design and updating processes. This literature describes that while the list of services included (or excluded) in an HBP is usually explicitly stated in the HBP design process, the decision criteria used and the actors involved in the process are often not transparently documented.^10 11^ A few studies clearly described the processes followed for the development and/or revision of the benefits package. For example, Thailand employed explicit priority setting processes, that is, cost-effectiveness, budget impact analysis, health systems capacity, equity, and ethical considerations, for revising the benefits package under the Thai Universal Health Scheme.^12^ The Netherlands uses necessity, effectiveness, cost-effectiveness, and feasibility (including affordability) as the key criteria for uptake of new provisions in its benefits package.^13^ Iran used evidence-based deliberative processes (EDPs) to revise its benefits package in 2021, in consultation with various stakeholders including clinicians, health economists, epidemiologists, service providers, patient groups, etc. The key criteria considered were categorised into three broad groups: quality of care (clinical evidence and safety), necessity (disease burden and severity, out-of-pocket expenditure), and sustainability (cost-effectiveness analysis, budget impact, pricing).^14^ Likewise, Kazakhstan also employs EDPs to update its list of benefits packages by considering key criteria such as severity of disease, financial burden for households, social priorities, effectiveness, cost-effectiveness, and budget impact.^15^

Economic evidence is widely used for policy-level decision-making in high-income countries.^16^ For instance, the National Institute for Health and Care Excellence (NICE) of the UK makes use of locally generated health technology assessment (HTA) evidence to inform decisions around intervention inclusion and price negotiations.^17^ Other developed nations like France and Australia also use local economic evidence to support pricing and reimbursement decisions.^18 19^ Like NICE, the HTA in India (HTAIn) has been established and is responsible for generating HTA evidence.^20^ Over the years, there has been an increase in the evidence base of studies on the cost-effectiveness of healthcare interventions in India. There has also been an effort to improve the availability of cost information.^21–23^ However, whether and to what extent this evidence is used to shape decisions under PM-JAY are not well documented.

The present study was undertaken to review the use of economic evidence in PM-JAY decision-making. It was designed to understand the role of evidence, processes, and various stakeholders in designing the initial HBP list and the basis of further revisions under PM-JAY and specifically assess the extent of the use of cost, cost-effectiveness, and/or HTA evidence for designing and pricing of HBP by the NHA. In addition, we also assessed the capacity of staff at NHA and SHAs to use the economic evidence for policy decisions in the context of PM-JAY.

## Methodology

### Study design

A mixed-methods study design was adopted with two specific components. First, in-depth interviews (IDIs) of stakeholders were conducted to understand the processes and mechanisms that went into designing and updating of the HBP and setting up of reimbursement rates under PM-JAY. This was supplemented by a document review. Second, a survey was undertaken involving staff at the NHA, SHAs, and other relevant stakeholders (engaged in the implementation and/or management of PM-JAY) to obtain a broad overview of the knowledge, attitude and practices related to the use of economic evidence in the context of decision-making for PM-JAY.

### Development of data collection tools

An exhaustive interview tool guide for the IDIs was prepared based on the various discussion meetings among the study authors (see online supplemental appendix 1). The tool guide was pilot tested by interviewing two of the authors who were involved in the design and revisions of the HBPs. The key themes included in the guide were aimed at understanding the process adopted in developing the initial HBP list and reimbursement rates, the rationale for revisions, the type of stakeholders involved, the type of evidence considered for inclusion under HBP and setting up of reimbursement rates. In addition, one of the themes specifically focused on the capacity and training needs of the staff at NHA and SHA related to the use of economic and HTA evidence within the PM-JAY ecosystem.

10.1136/bmjgh-2024-015079.supp1 Supplementary data

For the survey, a structured questionnaire focusing on the use of economic and/or HTA evidence and the use of cost data for setting up of reimbursement rates was prepared (online supplemental appendix 2). The questionnaire was pilot tested on a sample of 10 purposively selected respondents comprising of four SHA officials and six health economics researchers. Within the questionnaire, the first section was related to actual practices including the role of various stakeholders, key criteria and key evidence used during HBP revision, and formulation of reimbursement rates. The second section involved rating the importance of various stakeholders, factors, and sources of evidence in the HBP revision process on a Likert scale of ‘1–5’, where ‘1’ indicated least important and ‘5’ indicated extremely important based on individual perspectives.

### Participant recruitment and data collection process

For IDIs, the respondents were chosen purposively to include key individuals who had a role in designing or updating the HBP list and in setting or revising reimbursement rates. Three specific sets of participants were interviewed—current staff involved in updating of the HBP list and reimbursement rates; former senior-level officials of NHA involved in the initial design phase of HBP; and stakeholders from various development partners and technical support agencies who had been or were currently involved in providing technical support to the PM-JAY decision-making process. The interviews were conducted virtually during April and May 2022, and each interview lasted between 30 and 60 min. To supplement the information obtained from the IDIs, a web-based review of publicly available documents was undertaken. We searched the NHA’s official website which provides the most accurate and up-to-date documents related to HBPs. A total of 21 documents related to or elaborating the HBP development and revision process were reviewed. These included annual reports (n=3), user guidelines and HBP master documents (n=12), process flow documents outlining the constitution of various committees (n=4) and press releases (n=2).

The survey questionnaire was administered through two distinct approaches. First, an online version of the survey was sent to participants in webinar of the NHA online capacity building initiative ‘Nirantar Shiksha*’* for staff at NHA, SHA, and other relevant stakeholders.^24^ As the response rate from the webinar participants was low, the survey was then administered in a paper-based format to participants in an NHA workshop for officials and staff from NHA, various SHAs, and other stakeholders from development partners.^25^ The dual strategy aimed to maximise participation and gather valuable information from a wide range of stakeholders.

### Data analysis

All the IDIs were recorded for both audio and video. To maintain anonymity and confidentiality, unique codes were assigned to the interview videos and transcripts, the access to which was limited to the study authors. The anonymised and aggregated statements of the respondents from the IDIs were transcribed and analysed to draw final inferences. Thematic analysis of the data was conducted, focusing on overarching themes related to the HBP design process, the incorporation of economic evidence in HBP design and the staff’s proficiency in using economic evidence for HBP design. Facilitating this process, a comprehensive coding frame was developed. Two authors (DS, ASC) independently read all the transcripts to provide codes in response to the different sections in the transcript and identify the key themes. The identified themes and associated excerpts were compiled for systematic analysis using MS Excel. A descriptive analysis of survey data was undertaken to report frequencies and percentages.

### Patient and public involvement

Patients or the public were not involved in the design, conduct or reporting of this research study.

## Results

### Respondent characteristics

A total of nine interviewees were initially contacted for IDI to share their experiences on development/updating of HBPs of which seven were finally interviewed. These included a mix of current (n=2) and former officials (n=2) from the NHA, and officials from developmental partners (n=3) affiliated with the NHA. The respondents were involved in either one or more of the three phases of HBP development (HBP 1.0, HBP 2.0–2.2 or HBP 2022) and a majority of them had previous experience of working in the insurance sector, either in public state-level schemes or private hospitals.

A total of 80 non-duplicate responses were received from the online (n=26) and offline surveys (n=54). Around 70% of survey respondents were employed at the SHAs (representing 24 states and 2 union territories out of a total of 28 states and 8 union territories) and the rest were from NHA (18%) and development partners (11%), as shown in table 1. The survey participants had multiple roles and responsibilities, with around 61% being involved in HBP formulation, 61% involved in empanelment of the hospitals (the process of selection of public and private hospitals to provide healthcare services using predefined criteria set by the NHA^26^) and 54% were associated with beneficiary management (table 1).

**Table 1:** Background characteristics of study respondents

Parameters	Survey respondents N (%)	In-depth interviewees N (%)
Organisation of employment		
State level	57 (71.3)	–
Central level	14 (17.5)	4 (57.1)
Development partners	9 (11.2)	3 (42.9)
Highest educational qualification		
Bachelor	12 (15.0)	–
Master	67 (83.8)	7 (100)
PhD	1 (1.2)	–
Roles and responsibilities as defined by the individual*		
Empanelment of hospitals†	49 (61.1)	4 (57.1)
Beneficiary management	43 (53.7)	–
Health benefit package (HBP) formulation	51 (63.8)	7 (100)
Formulating standard treatment guidelines (STGs)‡	18 (24.1)	4 (57.1)
Capacity building	49 (61.1)	4 (57.1)
Others	44 (55.0)	–

*Each respondent could have more than one role and responsibility.

†Empanelled hospitals are healthcare facilities/providers that are enrolled in the PM-JAY scheme.

‡STGs have been introduced for processing HBPs under PM-JAY as a key step towards delivering minimum standard of care as per existent norms, and preventing fraud and abuse under the scheme.

PM-JAY, Ayushman Bharat Pradhan Mantri Jan Aarogya Yojana.

### Processes followed for the designing and updating of HBPs

**Figure 2:**
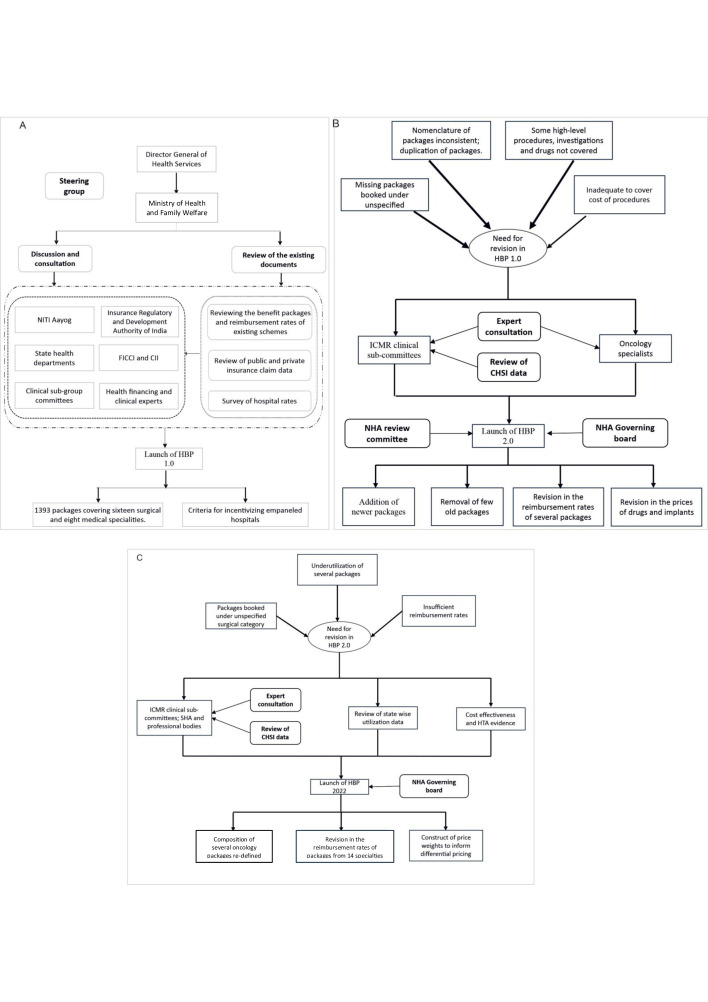
Process flow for designing and updating health benefit packages (HBPs): (A) HBP1.0, (B) HBP 2.0 and (C) HBP 2022. CHSI, Costing of health services in India; CII, Confederation of Indian Industry; FICCI, Federation of Indian Chambers of Commerce and Industry; HTA, health technology assessment; ICMR, Indian Council of Medical Research; NHA, National Health Authority; NITI, National Institution for Transforming India; SHA, State Health Agency.

Figure 2 summarises the process followed, the evidence used, and the stakeholder’s involvement in the designing and revising of the PM-JAY HBP list. The NHA was not institutionalised in the initial phase of development of the first draft of the benefits package (hereafter referred to as HBP 1.0). The Directorate General of Health Services of India initiated the process and constituted a technical committee to identify the list of services to be included in HBP 1.0 and the reimbursement rates. The technical committee comprised a diverse group of representatives from the Ministry of Health and Family Welfare, state health departments (particularly those with successful state insurance schemes), as well as health financing and clinical experts. This technical committee further constituted clinical subgroups who worked towards developing the speciality-wise list of packages (for HBP 1.0) by reviewing the benefits packages and reimbursement rates of existing schemes such as RSBY, CGHS, ESIS, railways and other state health insurance schemes. Important criteria considered for inclusion in the HBP 1.0 were disease burden, utilisation rates of the packages in the previous health insurance schemes of India and out-of-pocket expenditure incurred on various disease areas. The draft benefits package was sent to the National Institution for Transforming India (NITI) Aayog (policy think tank and advisory body of the Government of India), for further review and feedback. The NITI Aayog conducted various meetings with Department of Health Research-Indian Council of Medical Research (ICMR) clinical subcommittees; Insurance Regulatory and Development Authority of India; representatives from private associations and industry such as the Federation of Indian Chambers of Commerce and Industry and the Confederation of Indian Industry (see online supplemental appendix 3).

Following discussions with stakeholders, specific changes were incorporated into the draft HBP 1.0. Specifically, this included criteria for incentivising empanelled hospitals, considering factors such as the level of accreditation, location in the aspirational districts, and being a teaching institute. In August 2018, the NHA was established to oversee the implementation and monitoring of PM-JAY. Subsequently in September 2018, the HBP 1.0 was officially launched and comprised of 1393 packages covering 16 surgical and 8 medical specialties. These packages were reimbursed using a common national reimbursement rate for all empanelled providers. An additional incentivisation or increment above the national rate was provided to teaching hospitals (10%), for a hospital that had a quality accreditation (10–15%) by the national board, and for a hospital located in a metro city (10%) or aspirational district (identified by the Government of India as needing special attention based on certain socioeconomic indicators) (10%).

After the roll-out of HBP 1.0, the NHA received feedback from SHAs, empanelled hospitals, and other stakeholders who expressed dissatisfaction with the packages’ reimbursement rates. Furthermore, an NHA internal review process highlighted scope for improvement in the package construct and nomenclature, including concern about duplication of packages across specialties (figure 2B). To address these issues, HBP 1.0 was subsequently revised based on expert consultations with ICMR clinical subcommittees. These committees reviewed the cost estimates generated from a national-level costing study—Costing of health services in India (CHSI) and compared it with the CGHS rates and existing HBP 1.0 package rates.^21^ NHA also collaborated with experts from Tata Memorial Hospital (TMH) for reviewing the oncology-related package rates. Based on the expert recommendations, the NHA review committee and the governing board approved the second version of the HBP (HBP 2.0), which was introduced in September 2019. This revision encompassed the addition of some newer packages and removal of a few packages that were part of HBP 1.0 as well as the adjustment of some package rates to either a higher or a lower price. In addition, some packages were split into different categories to allow for variations in approach/treatment modality/aetiology/complication of the package. For example, a package of ‘lap/open cholecystectomy with/without exploration of the common bile duct (CBD)’ was split into four independent and specific procedures to be booked for a particular patient depending on lap or open cholecystectomy and with or without exploration of CBD.^27^

Following the release of the HBP 2.0, the NHA noticed that certain procedures were still not included in the existing HBP list, resulting in repeated claims for unspecified packages. Several packages were also found to be listed as state-specific packages. In addition, the SHAs, medical experts, and other stakeholders requested for the inclusion of additional new packages. To address these issues, a minor revision was carried out in November 2020, known as HBP 2.1. Like HBP 2.0, this revision was primarily driven by consultations with the ICMR speciality-wise committees, experts from TMH and other leading medical institutes. At this point of time, the NHA had constituted its own medical expert cell which also provided its recommendations. The release of HBP 2.1 involved addition of new packages and procedures, including the introduction of a package related to organ and tissue transplant. In October 2021, in a further revision (HBP 2.2), rates of 400 procedures were revised based on the findings of the CHSI study and stakeholder consultation. At this point of time, the NHA constituted a special technical review committee for costing, to deliberate on the costing evidence to finalise the package rates.

After the previous round of revisions, the NHA conducted a review of PM-JAY claims utilisation data in early 2022. The analysis revealed that several packages were underused relative to the burden of disease, while others were still being booked under the ‘unspecified’ surgical category, thus indicating the need to incorporate additional packages and procedures. Key stakeholders including SHAs, empanelled hospitals, and professional associations also raised concerns regarding the need for adjusting the reimbursement rates. The combined insights from the internal review and stakeholder feedback, along with the availability of new cost evidence from the CHSI study, emphasised the need for another major revision.

The HBP utilisation patterns and CHSI cost estimates were presented to the speciality-wise expert committees. In parallel, an analysis of the CHSI study data was undertaken to inform potential price weights for refining differential provider payment rates for different categories of hospitals.^28^ In addition, evidence from cost-effectiveness studies/HTA was also taken into consideration; however, this was limited to the packages within the oncology specialty. The refined HBP list, finalised by the speciality-wise committees, was then presented to SHAs, clinical experts, healthcare associations, and industry partners. The revised HBP (HBP 2022) was officially released in April 2022 comprising of 1949 packages. HBP 2022 also introduced the concept of differential pricing to account for structural variation in the cost of service delivery. A higher reimbursement rate was recommended for tier 1 and tier 2 cities, while the national reimbursement rate was used for all remaining cities. Similarly, to adjust for differences in the resource use according to the level of care, higher rates were set for tertiary care procedures as compared with secondary care procedures. This revision also identified the importance of instituting a more systematic and evidence-based approach to the HBP revision process, particularly the use of economic evidence, leading to the establishment of the health financing and technology assessment (HeFTA) unit at the NHA.^25^

### The extent of the use of health economic evidence

I very well remember they were still thinking pretty much in the clinical terms, not in economic terms.

IDI respondents indicated that no cost-effectiveness or HTA evidence was used during the development of HBP 1.0. Those who were involved or aware (n=3) informed that as such, no empirical evidence on the cost of healthcare delivery was used for setting the reimbursement rates.

The prices for HBP 1.0 were decided by the experts based on a review of prices of existing schemes such as CGHS, RSBY.

However, this changed for HBP 2.0 when the data from a nationally representative costing study (CHSI) became available and provided cost estimates for 855 PM-JAY packages for eight specialties. A cost-price differential analysis was also undertaken to identify packages with large differences between the PM-JAY rates and CHSI cost estimates (1online supplemental table 1).

This analysis revealed that nearly 42% of the HBPs had reimbursement rates that were less than 50% of the actual cost of services. Subsequently, based on the findings of the CHSI study and stakeholder consultations, rates for 61% of packages were increased by an average of 14 000 Indian rupees (450–165 000 Indian rupees), while rates for 18% of the packages decreased by an average of 6356 (ranging from 200 to 74 500 Indian rupees). Following these adjustments, only 20% of the total HBPs in HBP 2.0 now had a reimbursement rate that was less than 50% of the actual cost.^21^

We gave them [medical committees] certain ranges, let’s say what is the highest price offered across, all the state schemes, what is the lowest price you know which is being offered, what is the average price across different schemes and what is cost of the package as reported by CHSI study.

**Figure 3:**
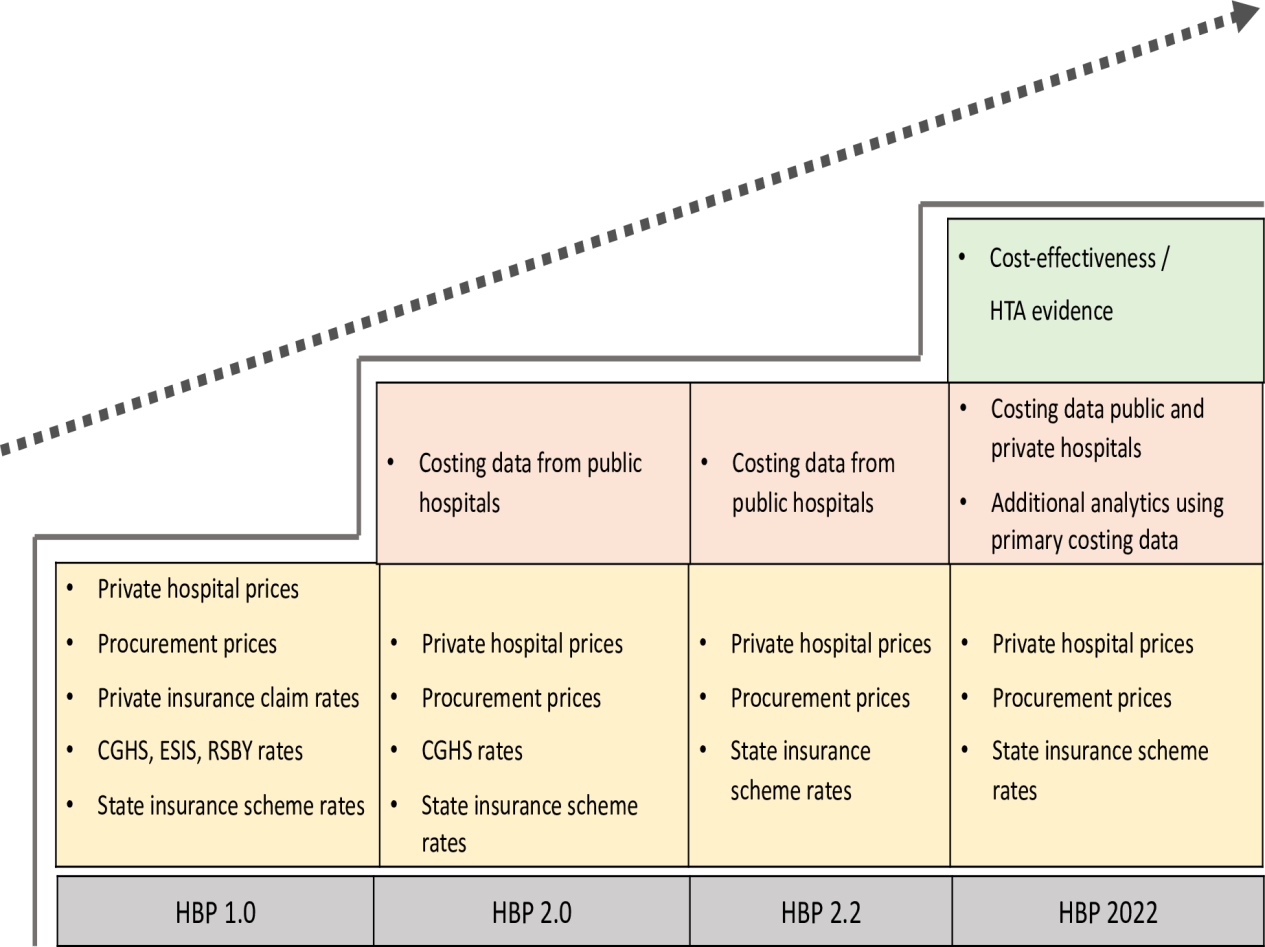
Health economic evidence used in different phases of HBPs. CGHS, Central Government Health Scheme; ESIS, Employees’ State Insurance Scheme; HBPs, health benefit packages; HTA, health technology assessment; RSBY, Rashtriya Swasthya Bima Yojana.

The prices of oncology drugs or implants enlisted in the National Pharmaceutical Pricing Authority or TMH procurement rate list were considered while revising the oncology-related package rates. Likewise, during the formation of HBP 2.2, reimbursement rates of 400 packages were also revised based on the findings of the CHSI study results. During the transitioning from HBP 2.0 to HBP 2.1, the focus was on addition of newer packages and was purely based on expert consultations (figure 3). No cost-effectiveness or HTA evidence was used during the transition from HBP 2.0 to 2.1.

During HBP 2022, the new cost data collected as part of the CHSI study (CHSI study at this point had generated additional cost information on the cost of healthcare delivery from private sector hospitals) was used to revise the reimbursement rates of packages from 14 specialties. Furthermore, price weights were constructed to inform differential pricing for the empanelled hospitals.^28^ It was hypothesised that various supply-side factors such as provider type (public tertiary, district and private hospitals), geographical location (rural, urban, metro, non-metro), and level of care (secondary, tertiary) could influence the cost of service provision. Based on the findings of the analysis from the CHSI data, a price weight of 15–17% for tier 2 cities and 15–25% for tier 3 cities, respectively, in addition to the base rate, was recommended. Similarly, a price weight of 10% and 25% for medical and surgical tertiary care packages was also recommended.^28 29^

Furthermore, this round of revision also marked the use of evidence from economic evaluations and adaptive HTA; however, this was limited to the packages within the oncology specialty. Specifically, the evidence was used to include or exclude services in the current list of HBPs or redefine the composition of existing packages and updating standard treatment guidelines.

### Survey results: perspective on stakeholders’ involvement and criteria for updating/pricing of HBPs

As the survey was administered during the formulation phase of HBP 2022, the findings of the survey based on the participant responses may reflect the processes followed during the time of development of HBP 2022.

**Figure 4:**
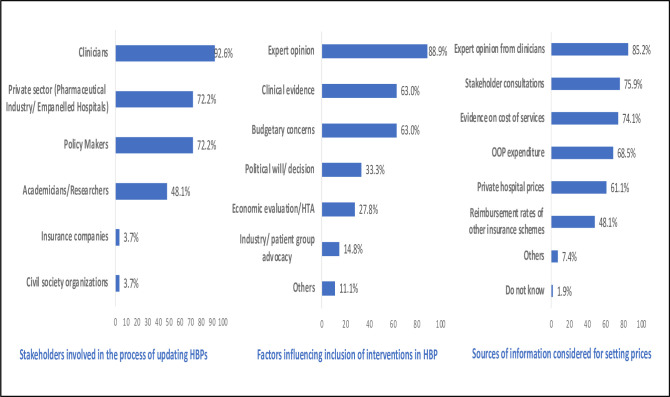
Survey findings based on actual practice: (1) stakeholders involved, (2) factors considered and (3) sources of information used for updating HBPs and their reimbursement rates. HBPs, health benefit packages; HTA, health technology assessment; OOP, out-of-pocket.

The majority of the survey participants identified clinicians as being consulted to inform the HBP revisions (92%). Private sector representatives and policymakers were each identified by 72% of the sample, while less than 50% reported academicians or researchers as being consulted in the HBP revision process (figure 4). Expert opinion (89%), clinical evidence (63%), and budgetary concerns (63%) were identified as factors influencing the selection of interventions as part of HBPs. Only 28% of the respondents identified cost-effectiveness evidence as a criterion being used in this process. In relation to information used, a majority (85%) of the respondents mentioned expert opinion from clinicians, followed by stakeholder consultations (76%) and cost evidence (74%), as sources considered for deciding the reimbursement rates. Clinical expert opinion (61%), followed by primary data collection and analysis (46%), secondary data analysis (33%), and published data (20%) were the primary sources of cost evidence used for deciding the reimbursement rates. When asked to identify the best source of costing evidence in the context of price setting, development of a national cost data repository (67%), followed by primary data collection by NHA/SHA were suggested by the survey respondents.

**Figure 5:**
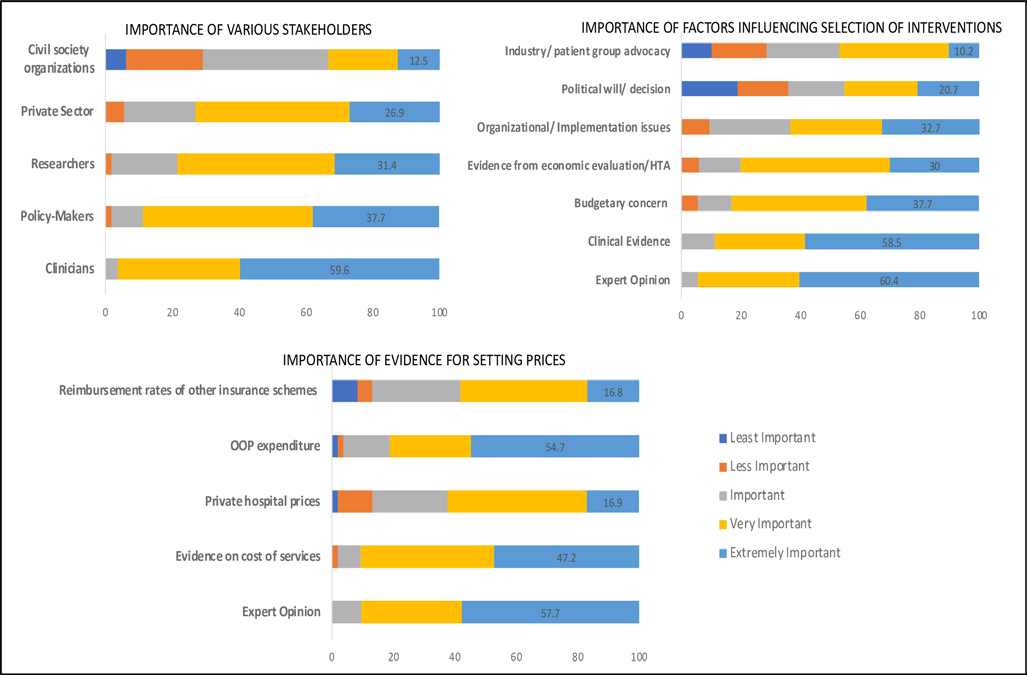
Survey respondents’ perspective on the importance of different stakeholders, factors influencing HBP selection and different sources of evidence in updating of HBPs and their reimbursement rates. HBPs, health benefit packages; HTA, health technology assessment; OOP, out-of-pocket.

Most of the survey respondents rated the role of clinicians (60%) followed by policymakers (38%) as extremely important in the context of developing/updating the HBP list. Researchers or academicians were considered extremely important by only 31% of the survey respondents. Likewise, most of the respondents rated expert opinion (60%) and clinical evidence (59%) as extremely important criteria for selecting interventions as a part of HBP. In contrast, budgetary constraints and evidence from economic evaluations or HTA were considered extremely important only by 38% and 30% of the respondents, respectively. Lastly, around 60% of the survey respondents rated expert opinion, followed by out-of-pocket expenditure estimates (54%) and estimates of healthcare delivery costs (47%) as important criteria for estimating reimbursement rates (figure 5).

**Figure 6:**
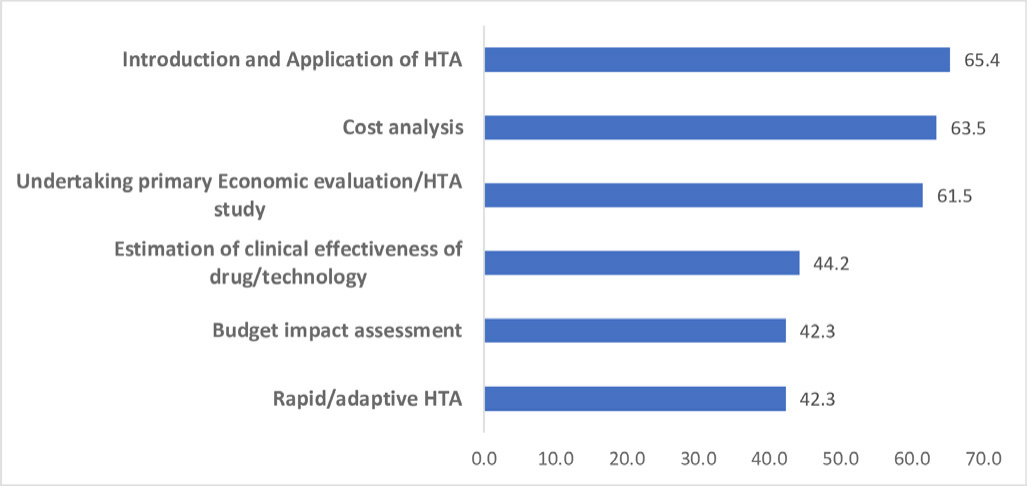
Survey respondents’ perspectives on areas needing capacity building. HTA, health technology assessment.

Training needs were also assessed in the survey and the respondents were asked to identify areas needing capacity building. Around 65% of the respondents selected courses on the introduction and application of HTA, followed by cost analysis (63.5%) and undertaking of cost-effectiveness evaluation (61.5%) as key areas for capacity building (figure 6).

## Discussion

This paper explores the processes and the role of economic evidence (ie, cost, cost-effectiveness and/or HTA evidence) that went into designing and pricing of packages included in PM-JAY HBP. Our findings suggest that the recent HBP revisions have made greater use of empirical economic evidence in comparison with the earlier versions, but there is still a lot of ground to be covered in relation to the application of cost-effectiveness or HTA evidence in HBP designing. Costing evidence from a variety of sources including the national-level costing (CHSI study) has played an increasing role. The role of HTA and cost-effectiveness analysis was introduced recently, and its application was also very limited even in the most recent phase of revisions. At the same time, the capacity to generate and use economic evidence is still limited and there exists a significant gap in the skills of NHA and SHA staff with regard to application of cost-effectiveness and/or HTA evidence.

It is widely recommended that decisions on HBP design should include HTA evidence comprising of the findings on clinical effectiveness, safety, cost, cost-effectiveness, budget impact analysis, etc.^30 31^ Benefits packages designed using economic evidence have been argued to provide the best value for money.^32^ Lack of coherence between the package design and resource availability for its implementation can jeopardise the sustainability of any health insurance scheme.^31 33 34^ In contrast, our survey respondents rated the importance of expert opinion as an important factor for selecting intervention as part of packages, while other crucial factors such as economic evidence and budgetary constraints were ranked lower. Further, for setting package rates, expert opinion from clinicians and stakeholder consultations were both seen to play a significant role and were considered more important relative to economic evidence. In the absence of good-quality research and data, this may be a more pragmatic choice, however, to ensure that experts take transparent decisions, it is important to make use of deliberative processes and ensure experts are uniformly informed with the best available evidence including economic evidence.^34^

Potential barriers or challenges faced during incorporating HTA or economic evidence into HBP at PM-JAY included the lack of availability of high-quality, timely HTA evidence and inadequate capacity to understand the use of HTA evidence. The capacity to analyse and use the available data within PM-JAY officials is also limited. Our key informants recognised the need to recruit trained health economists and HTA specialists at the NHA. They also mentioned that staff workshops on health economics could only serve a limited supporting function. Regarding the available evidence, the pace of HTA evidence generation does not yet match the need. The HTAIn, the regulatory body for the conduct of HTA studies in India, has completed only 26 studies while HBPs have over 1900 packages. At the same time, the capacity to generate and use economic evidence is still limited.

Such capacity constraints are not unique to India alone. Similar studies to assess the use of HTA evidence have been undertaken in other low/middle-income countries as well.^35–37^ In Nigeria, the researchers assessed stakeholders’ capacity needs and perspectives on the use of HTA and priority setting for Universal Health Coverage (UHC). The study concluded that HTA is a valuable tool for designing benefits packages, clinical guidelines and service improvement. However, the availability of local data to support such decisions was considered to be inadequate.^35^

While the lack of good evidence is a hindrance, this has not stopped the NHA from moving forward with efforts to systematically update and revise the benefits packages. The findings here provide important lessons for other countries on how processes can be initiated and therefore drive the change necessary to improve the evidence one step at a time. For example, following the launch of PM-JAY, the Government of India funded the nationally representative cost study to help inform package rates. Furthermore, efforts have been made to use HTA for designing HBP 2022 (although for now limited to specific oncology packages), and, more recently, a special unit—HeFTA—has been set up within the NHA to ensure the use of HTA evidence for making decisions that provide best value for money. Recognising the urgent need for expedited evidence synthesis in the context of PM-JAY, the HeFTA unit was established to accept nominations for HTA appraisal of proposed interventions for inclusion in the HBPs from a wide range of stakeholders, including both public and private organisations. Additionally, the HeFTA unit is envisioned to conduct horizon scanning of new HTA evidence, and evaluate its generalisability for application in the context of PM-JAY.

This is one of the first studies that explores the process employed in the development and revision of HBP, and the extent of the use of economic evidence for policy decisions in the context of PM-JAY. A diverse group of stakeholders were approached ranging from former officials, current officials, and other stakeholders (from development partners and industry), who provided technical support in PM-JAY decision-making, to have an idea of different viewpoints on the HBP development processes. Nonetheless, the small sample size for the IDIs and the limited documentation on the HBP design process mean we may have missed some insights and/or opinions.

## Conclusion

Economic evidence is recognised internationally as a critical tool for priority setting in publicly funded healthcare. Within the context of India’s PM-JAY, expert opinion has been a valued tool used for decision-making around the development of HBP design and the price setting. While expert opinion is also based on scientific knowledge and evidence, however, the explicit use of evidence-based deliberative processes was not observed. PM-JAY provides a classic case study using a stepwise approach to strengthen the process and gradually move towards more evidence-based processes with expert opinion supplemented with empirical evidence. However, rigorous efforts will be required to support this change. There is a need for regular, up-to-date cost data systems to inform HBP price revisions. The development of the HeFTA unit will be critical for including new packages in future HBPs. Lastly, political commitment is also crucial for the continued systematisation of these processes to achieve UHC.

## Data Availability

All data relevant to the study are included in the article or uploaded as supplemental information. All data are included as part of the manuscript or supplemental files.
