# Supplementary material for: Understanding the extent of economic evidence usage for informing policy decisions in the context of India’s national health insurance scheme: Ayushman Bharat Pradhan Mantri Jan Aarogya Yojana (PM-JAY)
Source: BMJ Glob Health. 2024 Jun 10;9(6):e015079. doi: 10.1136/bmjgh-2024-015079 (PMC11168173; doi:10.1136/bmjgh-2024-015079)
Supplement: Supplementary data [file bmjgh-2024-015079supp001.pdf]

Supplementary Material

Supplementary Table 1: The use of economic evidence in prioritisation of packages and informing their prices in PM-JAY

	**HBP 1.0**	**HBP2.0**	**HBP2022**
Pricing/cost	*"The prices for HBP 1.0 were decided by the experts based on a review of prices of existing schemes such as CGHS, RSBY."*<br><br>*"The DHR costing study was not complete so there was no empirical costing evidence at that time, however, a review of existing schemes and individual published costing studies for specialties such as cardiology, was undertaken to guide the HBP prices."*<br><br>*"At that time, reliable and valid cost data was not available. …… So cost data were minimal consideration in all this process. I'll tell you the exact, methodology was a review, of C G H S packages, E S I packages and also the, earlier scheme of RSBY".*	*"The expert committees were provided with current HBP 1.0 rate, CHSI rate and CGHS rate. The expert committee debated and reached on a final rate for HBP 2.0, which could either be same as the CHSI rate, increased rate or decreased rate"*<br><br>*"So, we gave them [medical committees] certain ranges, let's say what is the highest price offered across, all the state schemes, what is the lowest price you know which is being offered, what is the average price across different schemes and what is CHSI cost of the package."*	*"The CHSI study data was used because this time even the private sector data was available"*<br>*"We had evidence for public and private hospital from the costing analysis …… and they gave us a fixed cost that this particular package is having this much cost. …. After that, we compared it with parallel schemes running … like Meghalaya is having their own scheme, a state scheme. ….. ….. in that [expert] committee it was decided that which price need to be kept. The costing one or the CGHS one or, there has to be 10% increase in that, or there has to be 20% increase and decrease in that. …. Costing committee, they decided."*
HTA	*"I would say, back in 2017, the idea of HTA was just building up. …… I very well remember they were still thinking pretty much in the clinical terms, not in economical terms."*		*"..for oncology packages NCG-TMH have themselves conducted rapid HTA processes for medical oncology drugs. They undertake rapid HTA in 4-6 weeks given the limited budget. So HTA evidence was used for oncology packages but beyond that I don't think it was used"*<br>*"HTA evidence for high value interventions was referred to during specialty committee meeting such as proton therapy, and trastuzumab for breast cancer"*<br>*"We have selected few procedures. There were some chemotherapy procedures. There were some high- end drug procedures, the pricing of these procedures was set with the help of HTA. And for undertaking, they are hiring also, who will be working specifically for HTA."*

Supplementary Table 2: Staff capacity and training needs specifically related to Health Technology Assessment (HTA)

Understanding about HTA	*"HTA means X versus Y, the for a treatment or intervention, HTA tells you which one is better and cost effective or is right way to go about it. So this is what I understand about HTA, tell me if I'm wrong over here."*<br><br>"I'm frankly speaking, I have some brief understanding of what HTA is, yes. But yeah, but that thing was being utilized for framing the current package."<br><br>*"I have heard about HTA, but I would like to more know more about it. I would like to enrol for the courses at PGI as well. I have a little understanding which I've taken from Nirantar Siksha sessions, and I'm really interested to understand it better."*
Capacity to undertake HTA studies	*"I don't think anybody in NHA-HBP team is aware of what HTA is and how it can be used. NHA has people who have a background of hospital industry and many are not doctors or researchers. HTA is based on economics which have to be understood properly"*<br><br>*"No, any change in this direction is from PGIMER and NHA is actually adopting and learning sort of things."*<br><br>*"HTA itself takes a lot of time. If we have some HTA evidence already available especially in the Indian context that should be used. NHA doesn't have time for full HTA, however, rapid HTA studies should be undertaken."*
Training needs	*"Obviously, especially for the team which is working on HBPs training is needed on HTA because this is not a one-time activity. Will be more than happy to have trainings/workshops for HTA. Since we are going to set up a pricing division at NHA having training on HTA will be of great help to us"*<br><br>*"Obviously it can definitely be there. It has to be done. "*<br><br>*"I'd be more than happy to continue with your PGI courses as well. And if DHR comes up with any training workshop, I'm sure that all of my teammates would love to enrol for that and learn more about HTA. And as we are considering that, setting up a pricing institute here or something of that sort here, and, working towards health economics within NHA itself, So it'll be a great help for us."*<br><br>*"There should be a course on costing and economic evaluations in healthcare, with, practical case studies, some examples which emphasizes the importance of such, , such knowledge and use of such knowledge in the, PMJAY "*<br><br>*"Transition is very fast in NHA HBP team, HTA can't be taught in 2 weeks or a short course it comes with a lot of practice, one can have a pool of people with HTA background at least 1 expert with health economics background who can use HTA evidence."*<br><br>*"HTA involves a lot of calculations, that have to be understood properly. The transition is very fast in NHA, HBP teams. So, people come, they get trained, they leave. So, I mean HTA is not something that you can teach somebody in a crash course and they start doing it. It's not that. So it comes with lot of practice and, problem solving. So that's how, people become good HTA experts. So I think this is something, I mean, one can have, maybe a pool of people, or at least*

	one expert should be there, who has the background of h g Health economics and who can actually use" <br>*"There are a lot of trainings in the NHA for different things, people learn and they forget unless they practically apply it. So, there should be experts in the field of HTA employed at the NHA"*

Appendix 1: In-depth interview tool guide

Part I: General Information

1. Name
2. Organization
3. Highest educational qualification
4. Area of expertise
5. Are you currently working for/ with the NHA on AB-PMJAY HBPs Or in the past worked for/ with the NHA on AB-PMJAY HBPs?
   - Name of organization: NHA or Other (please specify name)
   - For what **time period** have you been working on/ or supported the HBP work?
   - What was your **role & designation** while working on/ or supporting the HBP work?
   - What were **your responsibilities** in relation to the HBP work?
6. Did you have any **previous experience** in the design, updating, pricing or implementation of health benefit packages for any other public/ private health insurance scheme (besides AB- PMJAY)? If yes, please elaborate.

Part II: HBP Process

7. Which **HBP phase(s)** did you participate in? Please mention all that apply.
   - HBP 1.0 (If yes, please go to Qs 8)
   - HBP 1.0-2.0
   - HBP 2.0-2.1 and 2.2
   - HBP 2.2-3.0

Note: If the interviewee responds yes to HBP 1.0, go to Qs 8. Otherwise, skip Qs 8 and go to Qs 9.

8. Can you please give a **brief overview of the design process** for HBP 1.0?

a. How was the HBP package developed? What was the process?
   - Which **stakeholders** were involved in the HBP 1.0 design?
   - How were the **stakeholders selected**?
   - What was the **role and responsibilities of the different stakeholders**?

- What were key areas of **differing views** related to HBPs between the stakeholders? How was consensus developed between the different stakeholders?

b. For which of the following HBP decisions was **evidence/ information collected**? Specify what was the evidence/ information reviewed while making these decisions? What were the sources of evidence/ information used (were any specific tools used)?
   - list of interventions to be included in HBPs
   - Prices of HBPs

c. Was a **prioritization process** used to select or exclude HBPs?
   - If yes, what prioritization criteria were considered while considering packages?
   - How/Why was this criteria selected
   - Were there any formal/informal procedures e.g., Rules, thresholds or laws that influenced this process?

d. How were the **prices decided**

9. For each of the HBP revisions you were involved in (i.e., 1.0 - 2.0, 2.0 - 2.1 & 2.2 and 2.2 - 3.0, can you please provide information on the following:

a. Why were the **revisions made**
b. A brief **overview of the process** followed for updating HBPs
c. Which **stakeholders were involved** in updating HBPs?
   - How were the **stakeholders selected**?
   - What was the **role and responsibilities of the different stakeholders**?
   - What were key areas of differing views related to HBPs between the stakeholders? How was consensus developed between the different stakeholders?
d. For which of the following HBP decisions was evidence/ information collected? Specify what was the evidence/ information reviewed while making these decisions? What were the sources of evidence/ information used (were any specific tools used)?
   - List of interventions to be included in HBPs,
   - Prices of HBPs
e. Was a prioritization process used to select or exclude HBPs?

- If yes, what prioritization criteria were considered while considering packages?
- How/Why was this criteria selected
- Were there any formal/informal procedures e.g., Rules, thresholds or laws that influenced this process?

f. How were the prices decided? Was any cost data used and how it was applied?

10. Are there **any provisions for revising decisions** about the HBP inclusions/exclusions and their prices once the list has been finalized? Please provide details.

Part III: Capacity on updating HBPs

11. Have you heard about health technology assessment (HTA)? Can you explain what this means to you?

12. Do you have an understanding of methods of HTA/ how to use evidence from HTA study? If yes elaborate – please tell us your level of understanding and if you have used this in your work and how?

13. Does your organization use health economic/HTA evidence for the development/pricing of health benefit packages? Now? In the past? Is this formalized in the form of a standard methodology/guideline?

14. Are there any future plans in systematizing the revision of HBPs using HTA evidence? If yes please elaborate.

15. Do you have an understanding of methods related to use of cost data for price setting? If yes elaborate – please tell us your level of understanding and if you have used this in your work and how?

16. In your opinion should there be specific courses/training workshops for the staff to be able to (a) generate/use HTA evidence (b)use cost data for price-setting. If so, what specific areas of training do you see as important?

17. Are you aware of HTAIn within Dept. of Health Research (DHR) and what are its functions? Do you think that HTAIn might help in the work of NHA? If yes, how can it help?

18. Any other comments

Appendix 2: Survey questionnaire: Assessment of attitudes and practices related to use of economic evidence in context of AB-PMJAY

About this survey:

The National Health Authority, PGIMER, Chandigarh and the Center for Global Development are conducting a survey to assess the attitudes and practices of the staff at the National Health Authority (NHA) and State Health Agencies (SHAs) on updating of health benefits packages with a focus on the use of economic evidence and Health Technology Assessment (HTA) in the context of AB-PMJAY.
In this context we request you to fill this survey. Please answer all the questions. Your responses will be considered confidential and will be used for implementation research purposes only.

Email*
____

1. Please specify the organization you are currently employed at:
   National Health Authority
   State Health Authority (specify state) ____________
   Others please specify ____________

2. Please select your highest educational degree:
   a) PhD
   b) Master's
   c) Bachelor's
   d) Diploma
   e) Others

   Please specify the subject area
   ____

3. What is your current designation and department?
   ____

4. What are your roles and responsibilities at your organization? (You may select more than one response if applicable)

a) Finance-Budget/Accounts/Audit/State releases
b) Empanelment of hospitals
c) IEC activities
d) Capacity building/training
e) Beneficiary management
f) Call center
g) Grievance management
h) Co-ordination with states
i) Development of health benefit packages
j) Pricing and deciding package rates

k) Formulation of standard treatment guidelines
l) Monitoring and Evaluation
m) Research
n) Claim settlement
o) Other:____________________________________

5. When deciding on a particular treatment or intervention for inclusion as a health benefit package (HBP), which of the following criteria is currently considered within <u>your</u> organisation/ context? (May select more than one options):
   a) Expert opinion
   b) Budgetary constraints
   c) Clinical evidence
   d) Evidence from economic evaluation/Health Technology Assessment
   e) Organizational/ Implementation issues
   f) Political will/ decision
   g) Industry/ patient group advocacy
   h) Don't know
   i) Other: ______________________

6. In <u>your</u> opinion, please rate on a scale of 1-5, the importance of each of the following criteria while deciding on whether to include a particular treatment/intervention as part of HBP (1 least important; 5 most important)
   a) Expert opinion

	1	2	3	4	5	
Least important	◯	◯	◯	◯	◯	Most important

   b) Budgetary constraints

	1	2	3	4	5	
Least important	◯	◯	◯	◯	◯	Most important

   c) Clinical evidence

	1	2	3	4	5	
Least important	◯	◯	◯	◯	◯	Most important

   d) Evidence from economic evaluation/Health Technology Assessment

	1	2	3	4	5	
Least important	◯	◯	◯	◯	◯	Most important

   e) Organizational/Implementation issues

	1	2	3	4	5	
Least important	◯	◯	◯	◯	◯	Most important

f) Political will/ decision

	1	2	3	4	5	
Least important	◯	◯	◯	◯	◯	Most important

g) Industry/ patient group advocacy

	1	2	3	4	5	
Least important	◯	◯	◯	◯	◯	Most important

7. In the process of developing/updating HBP who, among the following, were consulted in your context (You may select more than one option)

a) Clinicians
b) Policy-makers
c) Academicians/Researchers/Health economist
d) Private sector (Pharmaceutical Industry/ Empaneled Hospitals)
e) Civil groups
g) Don't know
h) Other: ____

8. In your opinion, please rate on a scale of 1-5 the importance of each of the following stakeholders in the process of estimation or updating HBP (1-least important 5-most important)

a) Clinicians

	1	2	3	4	5	
Least important	◯	◯	◯	◯	◯	Most important

b) Policy makers

	1	2	3	4	5	
Least important	◯	◯	◯	◯	◯	Most important

c) Academicians/Researchers/Health economist

	1	2	3	4	5	
Least important	◯	◯	◯	◯	◯	Most important

d) Private sector (Pharmaceutical Industry/ Empaneled Hospitals)

	1	2	3	4	5	
Least important	◯	◯	◯	◯	◯	Most important

e) Civil groups

	1	2	3	4	5	
Least important	◯	◯	◯	◯	◯	Most important

9. Which sources of information are currently considered in your context while setting prices of HBPs? Please select from the following (You may select more than one options):
   a) Expert opinion from clinicians
   b) Stakeholder consultations
   c) Evidence on cost of services
   d) Prices charged by private hospitals
   e) Out-of-pocket expenditure incurred by patients
   f) Reimbursement rates of other insurance schemes (CGHS, ESIS etc) (please specify if known)
   g) Don't know
   h) Other: ____

10. In your opinion, please rate on a scale of 1-5, the importance of each of the following criteria for estimating prices of HBP (1 least important; 5 most important)
   a) Expert opinion

	1	2	3	4	5	
Least important	◯	◯	◯	◯	◯	Most important

   b) Evidence on cost of services

	1	2	3	4	5	
Least important	◯	◯	◯	◯	◯	Most important

   c) Prices charged by private hospitals

	1	2	3	4	5	
Least important	◯	◯	◯	◯	◯	Most important

   d) Out-of-pocket expenditure incurred by patients

	1	2	3	4	5	
Least important	◯	◯	◯	◯	◯	Most important

   f) Reimbursement rates of other insurance schemes (CGHS, ESIS etc)

	1	2	3	4	5	
Least important	◯	◯	◯	◯	◯	Most important

11. What is the source of cost evidence used by your organization?
    a) Expert opinion
    b) Primary data collection and analysis
    c) Secondary data analysis
    d) Use of published data
    e) Other: ______________________

12. In your opinion, which among the following is the best source of costing evidence for NHA/SHA for the purpose of priority setting and or price setting in context of health benefit packages
    a) A national public repository having costing data should be developed
    b) NHA/SHA staff should collect primary data in accordance to a standardized methodology as and when required
    c) NHA/SHA should outsource collection of cost data in accordance to a standardized methodology to private agencies/research institutes
    d) Published data should be used
    e) There is no need of any data, expert advice should be sought
    f) Other: _______________________________________

13. Please rate your understanding of the application of the following concepts in HBP design and pricing:
    a) Use of Health Technology Assessment (HTA) evidence

Excellent understanding	Good understanding	Fair Understanding	No idea at all

    b) Use of costing evidence

Excellent understanding	Good understanding	Fair Understanding	No idea at all

14. In reference to health economics please select the areas where you need additional capacity building? (You may select more than one options):
    a) Overview of Health Economics
    b) Introduction and Application of HTA
    c) Undertaking primary Economic evaluation/HTA study
    d) Estimation of clinical effectiveness of drug/technology
    e) Cost analysis
    f) Rapid/adaptive HTA
    g) Budget impact assessment
    h) Other: ____

Thank you for participating in our survey. If you have any questions, please write to us at healtheconomics@pgisph.in

Appendix 3: Details of various organizations involved in formulation and revision of HBPs within PM-JAY

1. **National Institution for Transforming India (NITI) Aayog**

   NITI Aayog is a policy think tank of the Indian government providing both directional and policy inputs. The NITI Aayog plays an important role in designing the strategies for the long term policies and programmes put forth by the government of India. NITI Aayog also gives relevant advice to the centre and state governments as well as to the Union territories[1].

2. **Department of Health Research (DHR)**

   Department of Health Research (DHR) was created as a separate Department within the Ministry of Health & Family Welfare (MoHFW) by an amendment to the Government of India on 17th Sept 2007. The aim of the DHR is to bring modern health technologies to the people through research and innovations related to diagnosis, treatment methods and vaccines for prevention; to translate them into products and processes and, in synergy with concerned organizations, introduce these innovations into public health system[2].

3. **Indian Council of Medical Research (ICMR)**

   The Indian Council of Medical Research (ICMR), New Delhi, is the apex body in India for the formulation, coordination, and promotion of biomedical and health research, and is one of the oldest medical research bodies in the world. The ICMR has always attempted to address itself to the growing demands of scientific advances in biomedical research on the one hand, and to the need of finding practical solutions to the health problems of the country, on the other. It also sets the ethical guidelines for biomedical and health research in the country[3].
   The ICMR is funded by the Government of India through the Department of Health Research, Ministry of Health and Family Welfare. ICMR's 26 National Institutes address themselves to research on specific health topics like tuberculosis, leprosy, cholera and diarrhoeal diseases, viral diseases including AIDS, malaria, kala-azar, vector control, nutrition, food & drug toxicology, reproduction, immuno-haematology, oncology, medical statistics, etc. Its 6 Regional Medical Research Centres address themselves to regional health problems and aim to strengthen or generate research capabilities in different geographic areas of the country.

[1]NITI Aayog.About us. [Available from: https://niti.gov.in/objectives-and-features]
[2]Department of Health Research. About Department. [Available from: https://dhr.gov.in/about-us/about-department]
[3]Indian Council of Medical Research. About ICMR. [Available from: https://www.icmr.gov.in/aboutus.html

Sharma D, *et al. BMJ Glob Health* 2024; 9:e015079. doi: 10.1136/bmjgh-2024-015079

4. Directorate General of Health Services (DGHS)

Directorate General of Health Services (DGHS) is a crucial administrative arm operating under the Ministry of Health and Family Welfare (MoHFW). The DGHS serves as the apex technical authority responsible for shaping and implementing various health policies and programs across the country. It plays a central role in coordinating public health initiatives, managing healthcare infrastructure, and overseeing the deployment of medical services.[4]

DGHS, is actively involved in diverse health-related functions, including disease prevention, health promotion, and the regulation of medical practices. It works to ensure the availability and accessibility of quality healthcare services to the Indian populace. Additionally, DGHS is instrumental in managing human resources in the health sector, supporting the development of a skilled workforce to meet the evolving healthcare needs of the nation.

As a pivotal entity within the Indian healthcare system, DGHS collaborates with state health departments, central health agencies, and various stakeholders to address health challenges and promote overall well-being.

5. Insurance Regulatory and Development Authority of India (IRDAI)

Insurance Regulatory and Development Authority of India (IRDAI), is a statutory body formed under an Act of Parliament, i.e., Insurance Regulatory and Development Authority Act, 1999 (IRDA Act, 1999) for overall supervision and development of the Insurance sector in India[5].

The powers and functions of the Authority are laid down in the IRDA Act, 1999 and Insurance Act, 1938. The Insurance Act, 1938 is the principal Act governing the Insurance sector in India. It provides the powers to IRDAI to frame regulations which lay down the regulatory framework for supervision of the entities operating in the Insurance sector. Section 14 of the IRDA Act,1999 specifies the Duties, Powers and Functions of the Authority[6].

"The key objectives of the IRDAI include protecting the interest of policyholders, speedy and orderly growth of insurance industry, speedy settlement of genuine claims, effective grievance redressal mechanism, promoting fairness, transparency and orderly conduct in financial markets dealing with insurance, prudential regulation while ensuring the financial security of the Insurance market[7]."

[4]Directorate General of Health Services, MoHFW, India.Abou Directorate General of Health Services. [Available from https://dghs.gov.in/]
[5]Insurance Regulatory and Development Authority of India [Available from: https://irdai.gov.in/home]
[6]Insurance Regulatory and Development Authority of India. Duties, powers and functions of IRDAI. [Available from: https://irdai.gov.in/en/web/guest/duties-and-responsibilities]
[7]Insurance Regulatory and Development Authority of India. What we do? [Available from: https://irdai.gov.in/what-we-do]

6. Federation of Indian Chambers of Commerce and Industry (FICCI)

Established in 1927, FICCI is the largest and one of the oldest apex business organisations in India. "A non-government, not-for-profit organisation, FICCI is the voice of India's business and industry. From influencing policy to encouraging debate, engaging with policy makers and civil society, FICCI articulates the views and concerns of industry. It serves its members from the Indian private and public corporate sectors and multinational companies, drawing its strength from diverse regional chambers of commerce and industry across states, reaching out to over 2,50,000 companies.

FICCI provides a platform for networking and consensus building within and across sectors and is the first port of call for Indian industry, policy makers and the international business community[8]."

7. Confederation of Indian Industry (CII)

"The CII works to create and sustain an environment conducive to the development of India, partnering Industry, Government, and civil society, through advisory and consultative processes. CII is a non-government, not-for-profit, industry-led, and industry-managed organization, with around 9,000 members from the private as well as public sectors, including SMEs and MNCs, and an indirect membership of over 300,000 enterprises from 286 national and regional sectoral industry bodies.

For more than 125 years, CII has been engaged in shaping India's development journey and works proactively on transforming Indian Industry's engagement in national development. CII charts change by working closely with Government on policy issues, interfacing with thought leaders, and enhancing efficiency, competitiveness, and business opportunities for industry through a range of specialized services and strategic global linkages. It also provides a platform for consensus-building and networking on key issues.

Extending its agenda beyond business, CII assists industry to identify and execute corporate citizenship programmes. Partnerships with civil society organizations carry forward corporate initiatives for integrated and inclusive development across diverse domains including affirmative action, livelihoods, diversity management, skill development, empowerment of women, and sustainable development, to name a few[7]."

[8]Federation of Indian Chambers of Commerce and Industry. Industry's Voice for Policy Change. [Available from: https://www.ficci.in/api/home
[9]Confederation of Indian Industry. About us. [Available from: https://www.cii.in/About_Us.aspx.]

Sharma D, *et al. BMJ Glob Health* 2024; 9:e015079. doi: 10.1136/bmjgh-2024-015079
